# The study of early human settlement preference and settlement prediction in Xinjiang, China

**DOI:** 10.1038/s41598-022-09033-y

**Published:** 2022-03-24

**Authors:** Bo Tan, Hongwei Wang, Xiaoqin Wang, Suyan Yi, Jing Zhou, Chen Ma, Xinyan Dai

**Affiliations:** 1 College of Geographical Science, Xinjiang University, Urumqi, 830017 China; 2 Xinjiang Key Laboratory of Oasis Ecology, Xinjiang University, Urumqi, 830017 China

**Keywords:** Climate-change adaptation, Sustainability

## Abstract

When studying the human settlement process, it is of great significance to understand the prehistoric environment, economy and society by exploring the human–land relationship and the evolution of civilization reflected by the settlement environment. This paper explores the natural and social environmental preferences of early human settlements in Xinjiang, China, from the Palaeolithic to the Bronze Age (45 ka BP–2250 a BP). Through the characteristics of settlement preferences, the distribution of settlements is accurately predicted, and the relationship between settlement preferences and the evolution of the environment and civilization is verified and discussed. We summarize the needs and conditions of early human settlement from the perspectives of the social environment and natural environment and explain the stages, consistency and differentiation of the spatial and temporal evolution of settlement preferences with the interaction of adaptation and transformation. On this basis, we discuss the logical focuses and content of early human settlement preference research. This research provides a reference for the process, representation, driving mode, and research ideas of early human settlement preferences.

## Introduction

As the spatial relics of early human activities, settlement sites carry and record information about early human adaptation and transformation of the environment, interpreting and revealing the environmental information of settlement distribution can provide conditions for further understanding the interaction between humans and the environment during this period. Therefore, by studying the changing patterns of the distribution of human prehistoric sites and their geographical background, we can understand the adaptation and transformation processes of prehistoric humans to the living environment^1–4^, which are important topic in environmental archaeology research. Attempts to explain the relationships among prehistoric human settlement information, the development of early civilizations, and the natural environment date back to the 1950s^5–7^. These explanations allow us to use the past to further our understanding of the dynamic and complex systems in which humans interact with nature. Discussing the relationship between the spatial and temporal distribution of settlements and the geographic environment around the changing patterns of human prehistoric sites and their geographic context^1–3^, is an important environmental archaeological study issue. Studies of early human activities in Europe found that Neolithic settlers preferred Central European loess areas^8^, such as the so-called tribal areas in central and northern Germany^9,10^. A study of alpine sites in South Tyrol, Italy, reconstructed Mesolithic human settlement strategies and migration patterns in the alpine region at high altitudes^11^. In addition, over the past few decades, discussions of variable patterns and geographic contexts of human prehistoric sites have increasingly focused on interacting systems of ecological or livelihood patterns, and interactions with local–regional–global-scale tectonics, climates, or environments^12–18^.

Currently, the preference for human settlement in China is mainly based on the geographical and environment characteristics of site distribution, and the natural environment preference of prehistoric settlement sites in different regions and periods has been explored^10,19–23^, as well as social functional preferences^24,25^, explaining social domination, transportation, military defence, etc. The impact of sexual needs on settlement site selection reflects the obvious regional and epochal differences in human settlement. This evolution of human settlement preferences reflects the environmental constraints of early human settlement and the adaptation of human production to changes in human–land relations. However, the regionally distinct and asynchronous results revealed by different temporal and spatial scales suggest that case studies from more perspectives are needed to help us to understand the epochal characteristics and variability of human settlement in human–land interaction systems.

**Figure 1:**
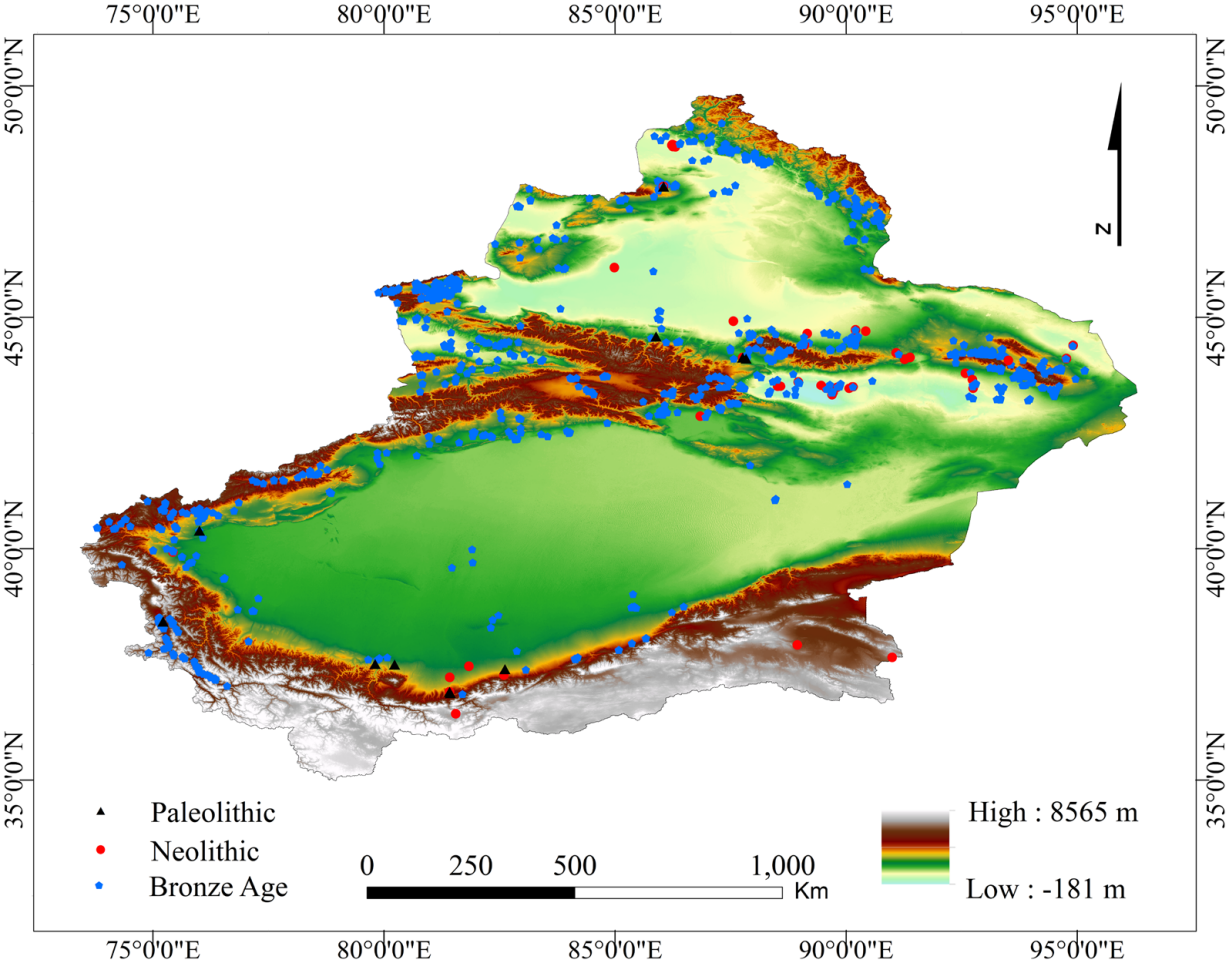
Location of the study area and distribution of settlements in different periods and altitude. The map was generated by ArcGIS 10.2, URL: https://support.esri.com/en/Products/Desktop/arcgis-desktop/arcmap/10-2-2#overview. The elevation data with a resolution of 30 m were obtained from the geospatial data cloud website (http://www.gcloud.cn).

Xinjiang is an important part of western China (Fig. 1), with a total area of approximately 1.66 million km^2^. After undergoing changes in a dry, wet, cold and hot Holocene climate^26–28^, a typical continental climate emerged. Mountains (Kunlun Mountains, Tianshan Mountains, Altai Mountains) and basins (Tarim Basin, Jungar Basin) in Xinjiang are staggered, and geographical landscapes such as mountains, deserts, glaciers, grasslands, woodlands, and the Gobi Desert, are widely distributed, with diverse temporal and spatial distributions. At the same time, Xinjiang is adjacent to Gansu and Qinghai in the east and Tibet in the south. It borders Mongolia, Russia, Kazakhstan, Kyrgyzstan, Tajikistan, Afghanistan, Pakistan and India to the northeast, north, west and southwest. Xinjiang is located in the central area where the northern Eurasian steppe culture overlaps with the Gansu–Qinghai cultural circle in northern China^29–33^. While colliding and communicating with surrounding cultures, Xinjiang’s prehistoric culture presents a mix of multicultural elements. Since the Holocene, the dry, wet, cold and hot climate change processes in the Xinjiang region^26–28^, have prompted corresponding changes in the natural environment, and its human culture is in a complex environment. Abundant cultural types have been born out of repeated adaptations and cultural exchanges^31^, making Xinjiang ideal for conducting environmental archaeological research. The current research on the prehistoric culture in the region has discussed cultural exchanges, livelihood patterns, climate and environmental change processes and their impacts of prehistoric humans^26,31–40^, but there have been fewer studies of behavioural preferences in settlement selection, and more emphasis has been placed on interpreting the decisive impact of the environment on human beings. The extraction and analysis of the geographical environment and social environment preferences of human settlement have been limited, and the research on the role of human subjectivity and agency has been insufficient.

In the prehistoric period, the inhabitants of this area created a brilliant historical culture and had cultural exchanges with the Central Plains and the surrounding areas. The early history of Xinjiang can be traced back to the late Palaeolithic. A large number of stone tools have been found on the western platform of Jiaohe Guchenggou in Turpan and at the Tong Tiandong and Buxel camel stone Palaeolithic sites. In the vast areas of the southern and northern Tianshan Mountains in Xinjiang, there are many fine stone and Neolithic remains from the Middle Stone Age. Researchers have unearthed fine stone, stone flakes and stone core stones, grinding plates, grinding rods, stone balls, grinding jade axes, jade adzes, and a small amount of coloured pottery and ceramic fragments with embossed and painted patterns^41–45^. From approximately 4500 a BP to 2250 a BP (Before Present), the oases in Xinjiang entered the Bronze Age and then the Early Iron Age^31,46–49^, and a large number of settlement sites from this period remain. The division of this time remains controversial, but this paper argues that the emergence and popularization of metal tools played two distinct roles in productivity and civilization progress, so this paper considers the time when the settlements entered the Iron Age, i.e., the time when bronze tools were widely replaced by iron tools, as the end of the Bronze Age.

This paper selects Xinjiang, China, as the research area; takes the human settlements (sites) between the Palaeolithic and Bronze Ages in Xinjiang, starting at nearly 45 ka, as an example to construct an early human settlement preference system; and explores the temporal and spatial characteristics of settlement preference. Based on the settlement preference characteristics, the Maxent model is used to predict the settlements in different periods to further understand the evolution of civilization in Xinjiang, to understand the relationship between the early human settlement process and the natural and social environment; and to provide a reference for the excavation and protection of sites in subsequent steps.

In summary, this article mainly answers the following questions:

- What are the characteristics of the environmental preferences for settlement in time and space?
- What are the relationships of settlement evolution and environmental evolution with civilization development?
- Are settlement predictions based on early settlement preferences feasible and credible?

## Results

**Figure 2:**
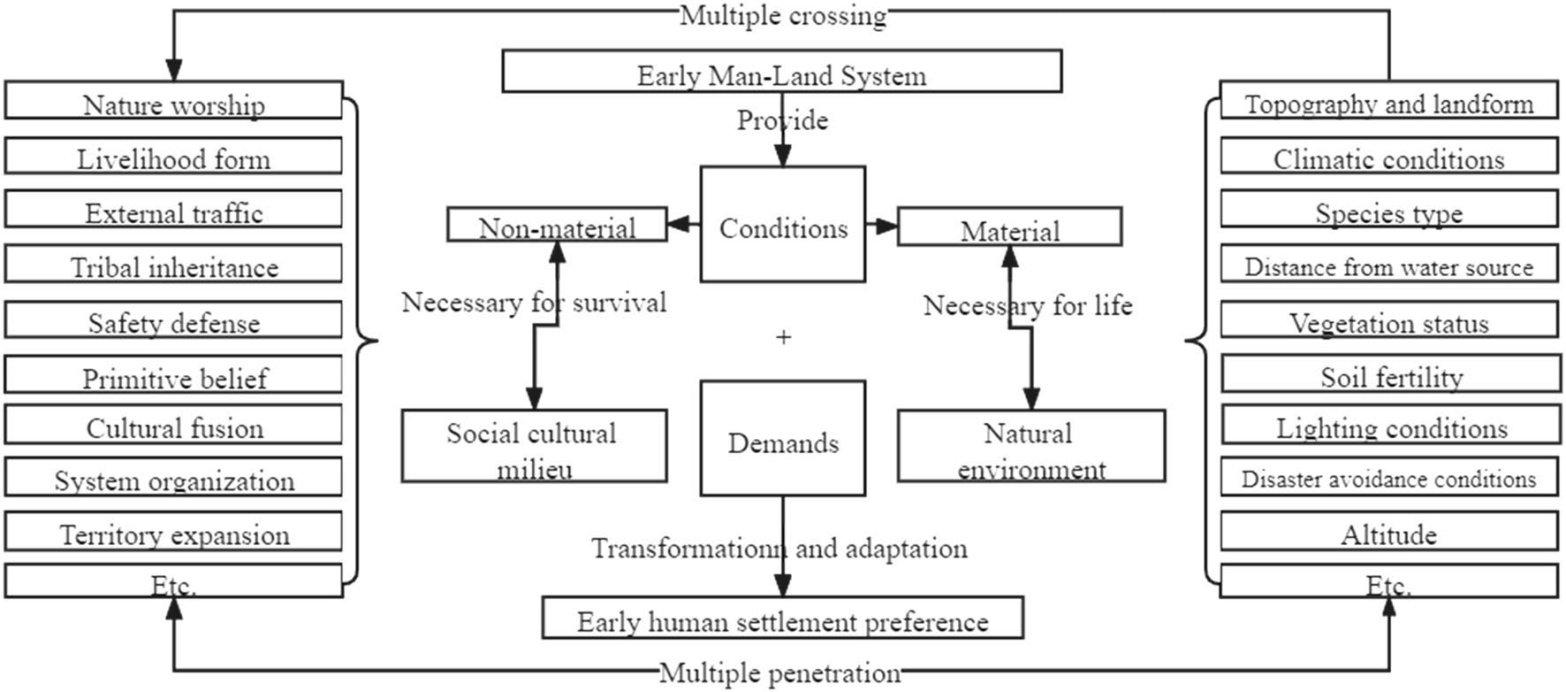
Composition and relationship of the early human settlement preference system.

This paper argues that under the existing early human-land system support conditions (natural environment support conditions and social environment support), a human settlement preference system is formed through the optimization of settlement environment conditions (Fig. 2). The settlement preference system is the product of human practice in the human–earth system, including the natural environment preference at the material level and the social and cultural preferences at the nonmaterial level. It is not the place where early human activities are performed. It is a secondary system composed of social behaviours established in the human–earth system. It is the generalization of a series of social behaviours produced by early human practice. It is influenced by human factors and natural environmental factors. From the perspective of human factors, there are needs such as early natural worship, livelihood forms, external transportation, tribal inheritance, security and defence, group cognitive guidance, cultural integration and diffusion, and the emergence of rights organizations^24–26,50–53^. These demands reflect the spiritual and cultural pursuit of early human social life, which is a necessary condition for life pursuit and constitutes social and cultural preferences through adaptation to the supporting conditions of the natural environment and the transformation of the natural environment. In terms of natural environmental factors, there are physical needs in the settlement process, such as suitability of topography and geomorphology, liveable climatic conditions, high biodiversity, proximity to water sources, a moderate number of vegetation types and moderate vegetation coverage, good soil fertility, abundant sunlight, a relatively low likelihood of disasters and suitable elevations^1,16,20,25,54,55^. Appropriate settlements are selected based on these needs, forming the natural environment preference of settlement activities.

### Geographical factor preference of settlement distribution in different periods

The study of the relationship between early human settlement and the geographical background is a key aspect of research on early human activities, covering the relationships of settlement activities with topography, the water system, regional structure, soil, climate and many other factors^1,3,56–58^. The geographical factors influencing early human settlement and life were clear and accompanied by temporal and spatial differences, and they contributed to the formation of and changes in early human culture^18^.

#### Preference characteristics of water source factors

The availability of water sources is an essential factor for human survival. The hydrophilic characteristics of early human settlement are obvious (Groucutt et al.^59^). The distance from a water source directly affects the convenience of water consumption and quality of life, and rivers are the most important water sources. Currently, there is no uniform classification standard for the distance from human settlements to rivers in early stages. In this paper, the distance from human settlements to rivers is divided into six levels: 0–1 km, 1–2.5 km, 2.5–5 km, 5–7.5 km, 7.5–10 km, and > 10 km.

**Figure 3:**
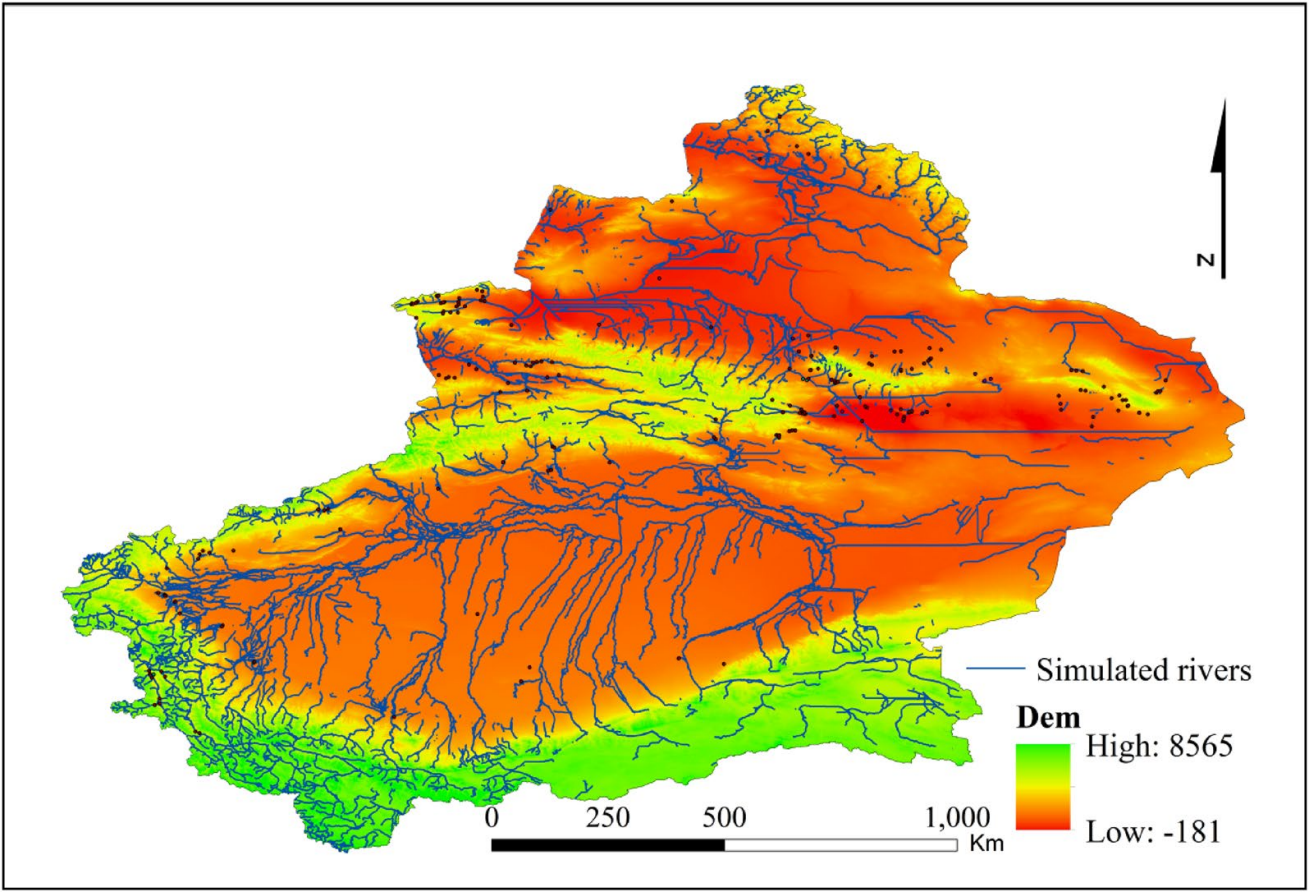
Simulation results of Xinjiang rivers. Map is created in ArcGIS 10.2.

The time span studied in this paper is 45 ka BP–2250 a BP, thus spanning much of the Holocene, and the time span is long. The alternation of cold and warm climatic conditions, dry and wet changes, and changes in topography have caused rivers to form, disappear, and change. Therefore, the distribution of modern rivers might not reflect the distribution of rivers in earlier periods, but the remnants of river changes still retain the characteristics of topographic depression, which can be effectively identified in DEM data. Therefore, this paper uses the hydrology tool set in ArcGIS software to extract the potential rivers in Xinjiang. Since the river extraction process changes with the size of the raster calculator extraction threshold^50^, this paper sets the threshold to 200,000 after attempting and integrating the extracted rivers with the data form modern rivers to simulate all possible rivers. The results are shown in Fig. 3.

**Figure 4:**
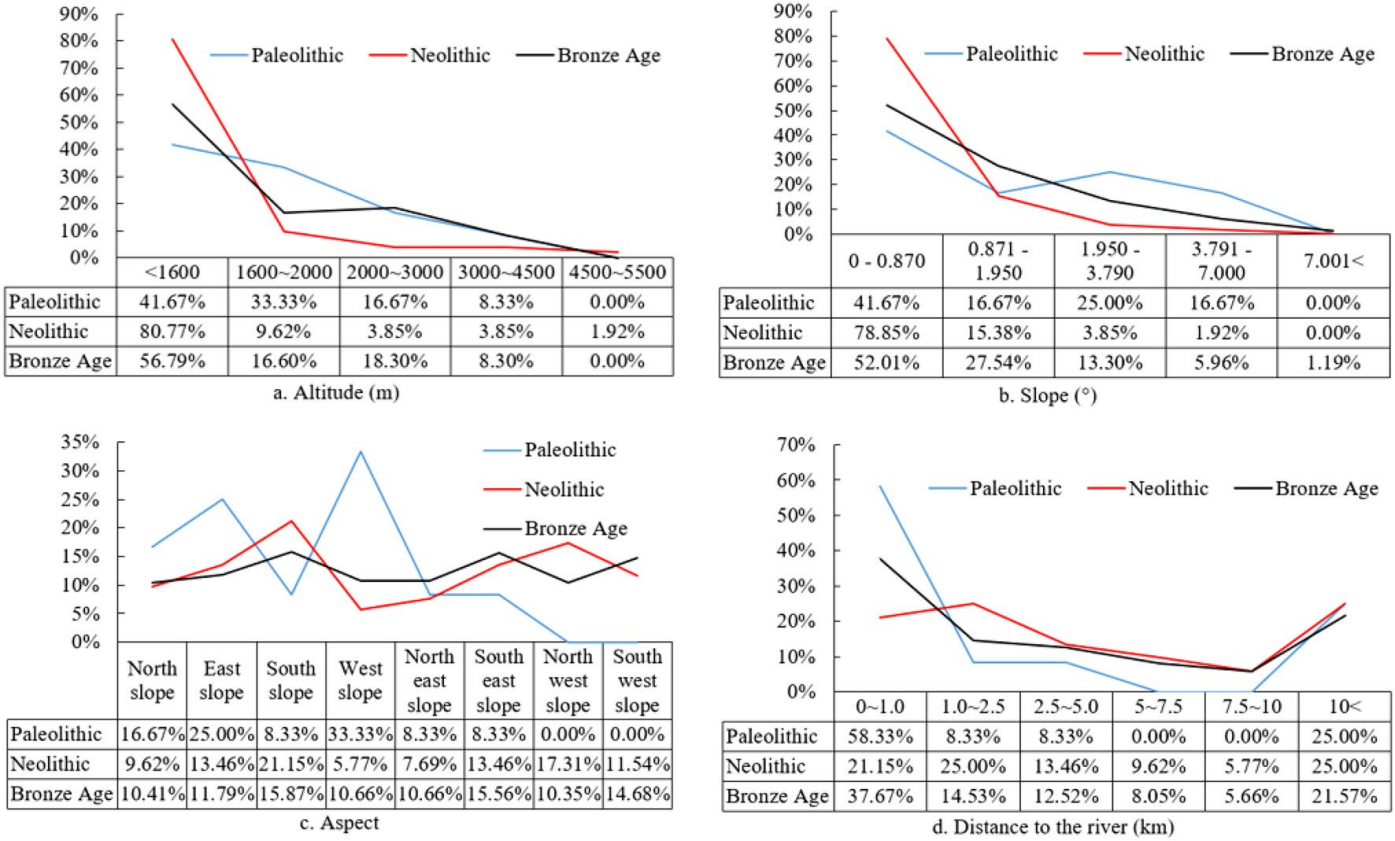
Topographic factors and water preference characteristics.

The statistical analysis of the quantitative relationship between settlements and river distance shows that (Fig. 4d) from the Palaeolithic to the Bronze Age, the settlement preference for water exhibits a ‘U’-shaped distribution. The settlements in the Palaeolithic and Bronze Ages are concentrated in the ranges is 0–1 km and 10 km from a river, with the Bronze Age (0.64) showing a higher correlation than the Neolithic (0.45). Compared with the Palaeolithic and Bronze Age settlements, the Neolithic settlements show a more diverse water preference.

#### Preference characteristics of topographic factors

The influence of elevation on human beings involves many factors, such as atmospheric physics, geochemistry and ecology. Among these factors, the low atmospheric pressure, low oxygen content, low temperature, low humidity and intense solar radiation affect the human body, with low oxygen content being the key factor. Extracting the elevation distribution of settlements in the study area, it is found that the early settlements are distributed between 132 and 4967 m, with the Palaeolithic settlements distributed between 1043 and 3163 m, the Neolithic settlements distributed between 46 and 4967 m, and the Bronze Age settlements distributed between 132 and 4155 m. Generally, at more than 2000 m above sea level, the human body begins to exhibit a hypoxia response. At more than 3000 m above sea level, the body’s oxygen dissociation curve begins to steepen, and hypoxia becomes more obvious. At more than 4500 m above sea level, the atmospheric pressure is close to half that at sea level, and the human body exhibits obvious hypoxemia, causing significant physiological reactions and a series of clinical problems. Humans cannot live long term at very high elevations above 5500 m^60^.

According to the aforementioned elevation range, the settlement elevation data of each period are classified and found (Fig. 4a). Compared with the Palaeolithic and Bronze Age settlements, the Neolithic settlements are concentrated in areas below 2000 m above sea level, accounting for 90.39%, which is nearly 20.00% higher than the previous two. The elevation-based concentration is higher, and the trend of settlements at low elevations is clear. However, in areas of severe hypoxia above 4500 m, there are Neolithic settlements (1.92%) but no settlements in the other periods. The proportions of Palaeolithic and Bronze Age settlements at 2000–3000 m and 3000–4500 m are significantly higher than those in the Neolithic, and the proportions are similar, which is interesting.

The slopes of the Palaeolithic and Neolithic settlements in the study area are between 0° and 7.00° (Fig. 4b). The slopes of the Bronze Age sites are between 0° and 13.47°. The proportion of settlements distributed between 0° and 0.87° (low slope) in each period is the largest, with Neolithic settlements having the highest proportion (78.85%).

The distribution of settlements on slopes with different aspects shows that (Fig. 4c) the distributions of Palaeolithic settlements on north-facing slopes, east-facing slopes and west-facing slopes are significantly greater than those on other slope aspects, with a cumulative proportion of 75%. The Neolithic settlements are preferentially distributed on south-facing slopes (21.15%), followed by northwest-facing slopes (17.31%), east-facing slopes (13.46%), and southwest-facing slopes (11.54%), with the remainder of the slope aspects accounting for less than 10%. The Bronze Age settlements are distributed on all slope aspects with small differences (range 5.52%) and a limited concentration on single slope aspect. The coefficients of variation (CVs) of the distribution of settlements in the Palaeolithic, Neolithic and Bronze Ages are 0.88, 0.38 and 0.18, respectively. The CV decreases gradually, indicating that the diversity of the distribution of settlements in the Palaeolithic to the Bronze Age increases and the dependence on the aspect decreases.

As shown in Table 1, Middle Palaeolithic settlements are mainly distributed in middle-elevation areas (dry alluvial plains, small undulating mountains, middle undulating mountains, and alluvial plains), with a cumulative proportion of 58.33%. Additionally, 16.67% are located in low-elevation lacustrine plains, 16.66% in middle-high elevations (alluvial plains, alluvial platforms), and 8.33% in high-elevation ice-water sedimentary plains. Neolithic settlements are mainly distributed in low-elevation alluvial plains (17.31%), low-elevation alluvial plains (13.46%) and middle-elevation dry alluvial plains (15.38%). Settlements are also distributed in low-elevation alluvial plains, low-elevation aeolian landforms, low-elevation dry alluvial plains, high-elevation alluvial plains, high-elevation hills and high-elevation undulating mountains, but the proportions of these categories are all less than 10.00%, and the preference characteristics are not obvious. Bronze Age settlements are distributed in all geomorphologic types except for high-elevation hills and mid-high-elevation undulating mountains, with the latter accounting for 12.35%. The CVs of settlements from the Palaeolithic to the Bronze Age in different geomorphic distribution ratios were 2.26, 1.69 and 1.22. The CVs decreased with time, similar to the pattern of the change in slope aspect of the settlements. The geomorphic types of settlements tended to be diverse and uniform, and the preference for special geomorphic types decreased over time.

**Table 1:** Preference characteristics of topography and geomorphological factors in different periods.

Landform type	Palaeolithic (%)	Neolithic (%)	Bronze Age (%)	Landform type	Palaeolithic (%)	Neolithic (%)	Bronze Age (%)
Low-elevation floodplain	0.00	17.31	7.81	Denuded mesa at middle and high elevations	0.00	0.00	0.06
Mid-elevation dry alluvial plain	25.00	15.38	4.54	Medium and high-elevation alluvial plains	8.33	0.00	2.27
Low-elevation floodplain	0.00	3.85	2.96	Medium and high-elevation alluvial platform	8.33	0.00	1.13
Low-elevation aeolian landform	0.00	1.92	0.19	High-elevation glacial water depositional plain	8.33	0.00	2.71
Low-elevation dry alluvial plain	0.00	7.69	2.08	High-elevation large undulating mountains	0.00	0.00	4.79
Low-elevation dry alluvial platform	0.00	0.00	0.06	Medium and high-elevation diluvial platform	0.00	0.00	0.06
Mid-elevation lacustrine plain	0.00	0.00	0.63	High-elevation small undulating hills	0.00	0.00	0.38
Mid-to-high-elevation alluvial plain	0.00	0.00	0.19	Mid-to-high-elevation and mid-range undulating mountains	0.00	1.92	12.35
Low-elevation lacustrine plain	16.67	3.85	0.76	Mid-elevation and mid-range undulating hills	8.33	0.00	0.00
Low-elevation hills	0.00	0.00	1.58	Mid-elevation glacial water alluvial plain	0.00	0.00	0.13
Mid-elevation alluvial plain	0.00	5.77	5.10	Low-elevation mid-range undulating mountains	0.00	3.85	0.13
Mid-elevation alluvial plain	8.33	5.77	7.62	Mid-elevation alluvial platform	0.00	0.00	0.69
Low-elevation floodplain	0.00	13.46	4.22	Low-elevation and small undulating mountains	0.00	0.00	0.38
Mid-elevation aeolian landform	0.00	0.00	0.76	High-elevation and large ups and downs	0.00	0.00	8.19
High-elevation diluvial plain	0.00	1.92	0.06	Low-elevation impact diluvial platform	0.00	0.00	0.95
Mid-elevation alluvial plain	0.00	5.77	8.07	High-elevation and extremely undulating mountains	0.00	0.00	1.89
High-elevation hills	0.00	1.92	0.00	Low-elevation lacustrine-diluvial plain	0.00	0.00	0.06
Ups and downs in high elevation	0.00	1.92	0.57	Extremely high elevation and large ups and downs	0.00	0.00	1.32
Mid-elevation hills	0.00	1.92	3.02	Medium-elevation small ups and downs	16.67	3.85	8.19
Modern glacier	0.00	0.00	0.06	Mid-elevation and mid-range undulating hills	0.00	1.92	4.03

#### Preference characteristics of soil type factors

The soil types of settlement distribution in different periods were statistically analysed (Table 2). Palaeolithic settlements were mainly distributed in the swamp soil area, accounting for 50.00%, followed by grey brown desert soil and saline soil and finally forest-shrub meadow soil and paddy soil. Each settlement featured a single soil type. In the Neolithic, settlements were distributed in 13 soil types, with the highest proportion (25.00%) in brown desert soil. The preference for temperate desert soil increased, while the preference for swamp soil decreased in the Bronze Age compared with the Palaeolithic. In the Bronze Age, the number of soil types associated with settlements increased again, spanning all soil types, and the preference for chestnut soil (also the dominant soil type in the Bronze Age) was much greater than that in the previous two periods.

**Table 2:** Proportion of settlements in different soil types.

Soil type	Palaeolithic	Neolithic	Bronze Age	Soil type	Palaeolithic	Neolithic	Bronze Age (%)
Other	–	1.92%	2.82%	Limestone	–	–	1.19
Meadow soil	–	7.69%	6.28%	Grey cinnamon	–	–	0.25
Straw soil	–	–	0.69%	Grey desert soil	–	7.69%	0.50
Chao soil	–	1.92%	3.14%	Grey brown desert soil	16.67%	–	1.32
Aeolian soil	–	11.54%	1.26%	Cold calcium soil	–	1.92%	5.71
Irrigated desert soil	–	5.77%	1.57%	Chestnut soil	–	9.62%	25.61
Irrigation silt	–	–	1.63%	Shrubby meadow soils	8.33%	5.77%	0.88
Cracked soil	–	–	0.06%	Paddy soil	8.33%	–	0.19
Permafrost	–	–	0.06%	New soil	–	–	0.06
Cold calcium soil	–	1.92%	–	Saline	16.67%	3.85%	1.95
Cold desert soil	–	1.92%	–	Swamp soil	50.00%	–	0.44
Chernozem	–	–	2.07%	Brown earth	–	13.46%	25.49
Black felt soil	–	–	0.63%	Brown desert soil	–	25.00%	16.20

### Preference characteristics of vegetation type factors

The distribution proportion of settlements in different vegetation type areas are shown in Table 3. Settlements in the Palaeolithic are concentrated in temperate semishrub and dwarf semishrub deserts (50%), followed by temperate cluster grass typical steppe (16.67%) and other types, but the proportions and total numbers were small. The Neolithic settlements were mainly distributed in cultivated vegetation (34.62%) and temperate semishrub-dwarf semishrub deserts (26.92%). Cultivated vegetation is the result of modern human transformation of nature, and cultivated plants are complex. Due to the promotion of cultivation techniques, the dependence of plant cultivation on the local climate is weaker than that of natural vegetation, and it does not have regional typicality. Therefore, the settlement preference of cultivated vegetation is not discussed. In addition to the above two vegetation types, settlements were also distributed in the other 12 vegetation types, but the proportions were less than 10.00%. The settlements in the Bronze Age were distributed in each vegetation type, but they were mainly concentrated in cultivated vegetation (17.90%) and temperate semishrub-dwarf semishrub desert (22.94%). The distribution proportions in temperate clustered grass typical steppe and temperate clustered grass-dwarf semishrub desert steppe were also greater than 10%. From the Palaeolithic to the Bronze Age, the distribution of settlements in the temperate semishrub-dwarf semishrub desert was always maintained at a high level, and the settlement preference for the two was strong. Additionally, the vegetation types of settlements were diverse.

**Table 3:** Proportion of settlements in different vegetation types.

Vegetation type	Palaeolithic	Neolithic	Bronze Age	Vegetation type	Palaeolithic	Neolithic	Bronze Age
Cultivated vegetation	–	34.62%	17.90%	Temperate steppe shrub desert	–	–	0.19%
Subalpine deciduous broad-leaved shrub	–	–	0.95%	Temperate dwarf grasses, dwarf semishrubs, desert steppe	8.33%	1.92%	15.37%
Temperate annual herbaceous desert	–	1.92%	0.69%	Other	–	3.85%	4.41%
Temperate deciduous sparse forest	–	3.85%	–	Temperate dwarf semiarbor desert	–	1.92%	0.13%
Temperate deciduous broad-leaved forest	–	3.85%	0.19%	Temperate semishrub, dwarf semishrub desert	5–	26.92%	22.94%
Temperate deciduous shrub	–	–	0.06%	Sparse alpine vegetation	–	–	0.19%
Temperate grasses and weeds in saline meadow	8.33%	9.62%	3.40%	Temperate grasses, cares and weeds swamped meadows	–	–	0.32%
Temperate grasses, weeds, meadow grassland	–	–	1.26%	Cold temperate and temperate mountain coniferous forests	–	–	0.50%
Alpine swamp	–	–	1.32%	Temperate tussock grass typical steppe	16.67%	1.92%	11.97%
Alpine grass, Carex grassland	–	1.92%	2.96%	Cold temperate zone, temperate swamp	–	1.92%	–
Temperate shrub desert	8.33%	1.92%	5.36%	Alpine Kobresia, weedy grass meadow	8.33%	–	1.51%
Temperate succulent saline dwarf semishrub desert	–	–	2.27%	Temperate grasses and weeds meadows	–	–	2.65%
Alpine cushion vegetation	–	–	0.06%	Alpine cushion-like shrub desert	–	3.85%	3.40%

### Traffic factor preference characteristics of settlement distribution in different periods based on traffic route simulation

Guided by the idea of constructing the resistance surface in the minimum resistance model, this paper simulates the early human traffic routes in Xinjiang with the early traffic route simulation method developed by Zhu et al.^50^, and the specific operation process is shown in Ref.^50^. The simulation factors used in this study include distance from a river, slope, vegetation, elevation and profile curvature. The resistance surface of the simulation factors was constructed, and the resistance level was divided. The significance of the first four simulation factors for traffic routes has been discussed^50,59–61^, and this article does not focus on them. Section curvature affects the acceleration and deceleration of flow during runoff, while in the early period when human social productivity was limited, transportation routes were mainly formed based on natural topographic features. Therefore, this study suggests that a change in surface profile curvature can also produce a change in the traffic route profile curvature, and the movement of people along the traffic route is also bound to be affected by increases or decreases in the profile curvature. The greater that the profile curvature is, the more obvious that the acceleration is, and the greater that the traffic efficiency is. Under similar conditions, people will choose high-efficiency routes. At the same time, the sectional curvature has a wide range of applications in soil and water conservation. The greater that the sectional curvature is, the greater that the topographic relief is, and the greater that the topographic change is. Soil erosion and landslides are more likely to occur in areas with high relief, and there is a possibility of disrupting the traffic route. Therefore, the division of the resistance of the sectional curvature must consider these dual effects^62–64^.

**Figure 5:**
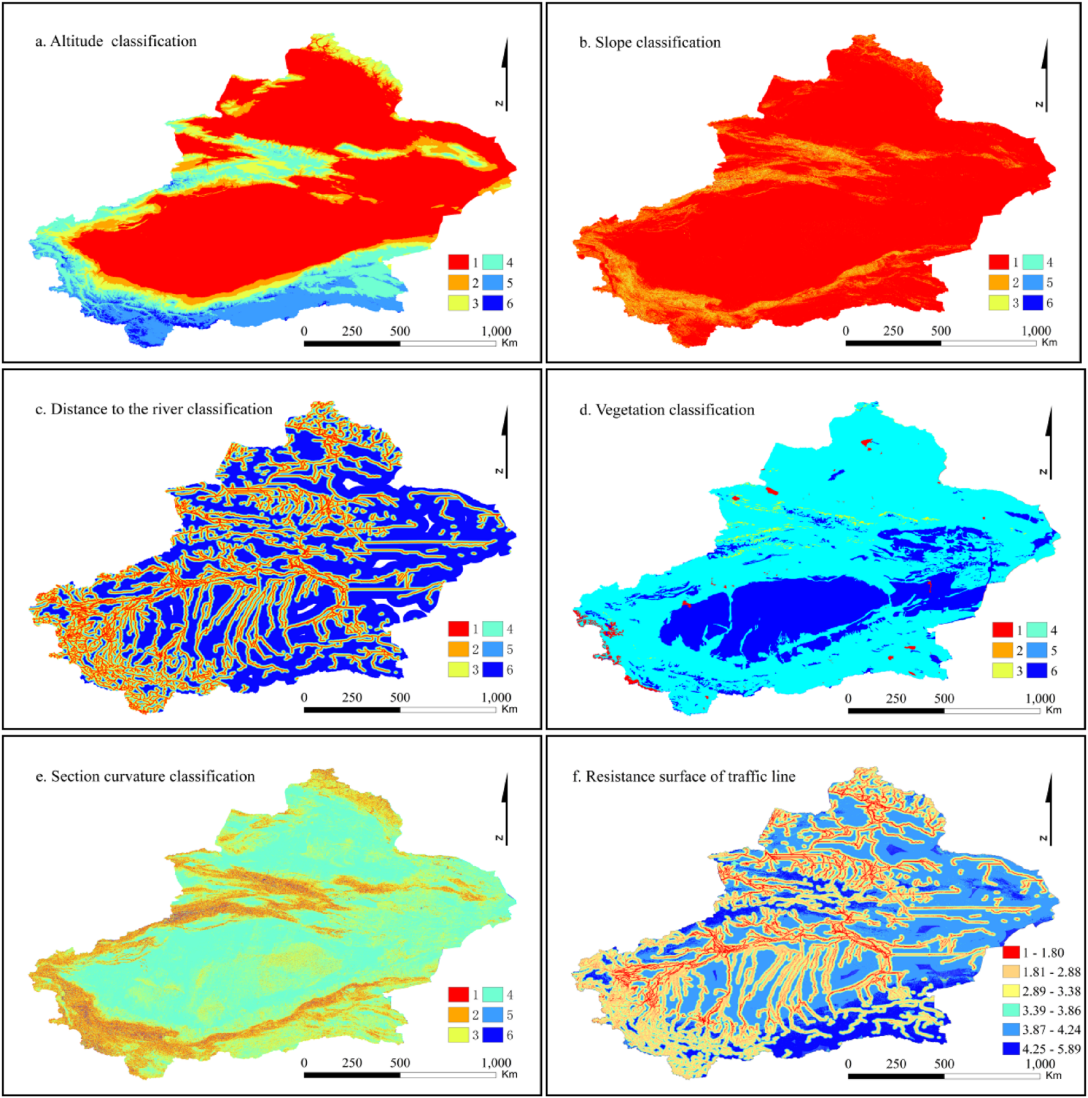
Resistance classification of traffic simulation factors (**a–e**) and simulation results (**f**). Maps are created in ArcGIS 10.2.

In this study, the resistance of traffic simulation factors is divided into 6 levels, i.e., 1–6 (Table 4). The classification is based on the literature^50,59,60,63,65^, in which the vegetation type classification is divided by the proportion of settlements. The greater that the profile curvature is, the higher that the traffic efficiency of people is, and the smaller that the resistance is. However, the possibility of blocked traffic routes should also be considered. Therefore, the classification of resistance levels should be based on the idea of moderately low resistance and high resistance at both ends. The higher that the factor resistance level is, the stronger the resistance is, and the more that unsuitable the traffic route is. The reclassification results of factor resistance according to the classification level in Table 2 are shown in Fig. 5a–e. The weight of each factor is estimated by an analytic hierarchy process, and then the resistance surface of the traffic route is obtained by grid calculation. The smaller that the resistance value is, the more likely that the path is to become a route. The lines with existing settlements are selected with the using the location tool, and their importance is calculated according to the index range value of the screened routes. Considering the consistency and feasibility of the simulation results, the range that accounts for less than 70% of the resistance value of the simulated routes (with resistance values between 1 and 1.8) is defined as the final route. The results are shown in Fig. 5f.

**Table 4:** Analogue factor classification and assignment.

Elevation (m)	Slope (°)	River (km)	Vegetation type	Section curvature	Grade
< 1600	0–0.870	0–1.0	Temperate semishrub, dwarf semishrub desert	> 18.3744	1
1600–2000	0.871–1.950	1.0–2.5	Cultivated vegetation	2.0896–18.3744	2
2000–3000	1.950–3.790	2.5–5.0	Temperate tussock grass typical steppe	1.2250–2.0896	3
3000–4500	3.791–7.000	5–7.5	Temperate grasses, weeds, saline meadows, temperate dwarf grasses, dwarf semishrub desert grasslands	0.6485–1.2250	4
4500–5500	7.001–13.470	7.5–10	Temperate shrub desert	0.2162–0.6485	5
> 5500	> 13.14	> 10	Other	0–0.2162	6

**Figure 6:**
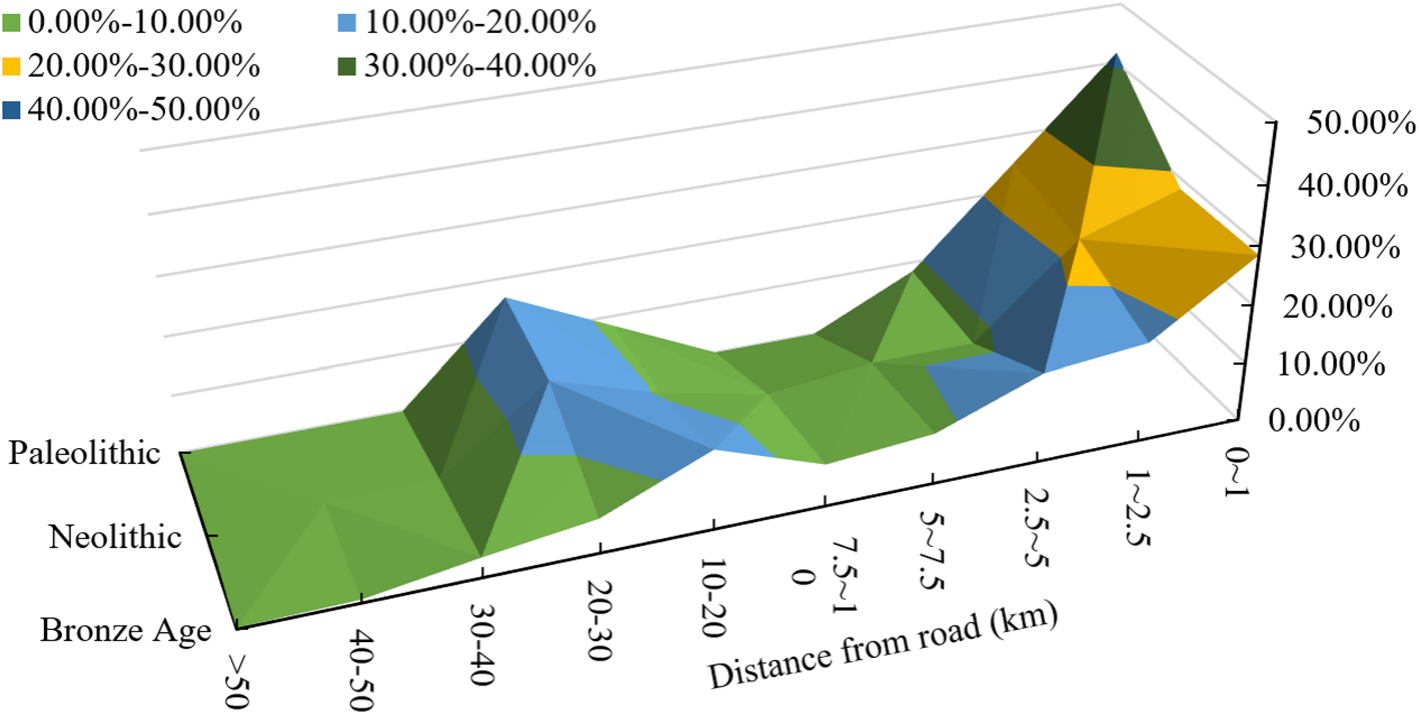
Distribution ratio of settlements at different river distances.

Taking 1, 2.5, 5, 7.5, 10, 20, 30, 40, and 50 km as the distance intervals, the multiloop buffer analysis of the simulated traffic routes is performed, and the preference of the settlements for traffic routes is assessed (Fig. 6). The settlement activities in the Palaeolithic, Neolithic and Bronze Age were mainly concentrated within distances of 0–2.5 km from a traffic route (the proportions were 55.33%, 52.94% and 45.37%, respectively). In addition, 1/3 to 1/4 of the total settlements were distributed in the range of 10–50 km. In the range of 20–30 km, the distribution of Palaeolithic settlements was 16.67%. In total, 15.09% of Neolithic settlements were distributed at distances of 30–50 km from a road. The distribution proportions of the Bronze Age settlements in the ranges of 10–20 km and 20–30 km were 13.49% and 5.83%, respectively. There was also a small number of settlements in the range of 30–50 km, and there were three settlements in the range of more than 50 km. From the Palaeolithic to the Bronze Age, the CV values of the distribution of settlements at different distances were 1.33, 0.89 and 0.83, indicating that the traffic preferences of human settlements tended to be diverse. The distribution range of settlements gradually expanded, and the spatial range of human activities increased. However, the traffic preference of settlements was still less than 2.5 km.

### Characteristics of cultural development preference of settlement distribution

#### Screening of central settlements and quantification of cultural radiation areas

Christaller’s central place theory points out that the concentration of settlements is an important indicator of the level of social development^66^, and central settlements exert clear spatial diffusion and attraction effects on surrounding settlements. In early human society, the cultural impact of central settlements on surrounding settlements and the spread of technology formed spatial radiation areas with a certain range, and the development, size and population of the central settlements dictated the magnitude of the cultural radiation capacity^51,67–71^. As a measurement of settlement development, the site scale is of great significance for retrieving and reconstructing the intensity of human activities and social output at a given time^53,69^. Therefore, this paper refers to the existing research on the scale division of domestic settlements^54,72–74^, dividing settlements by size as 0–1000 m^2^, 1000–5000 m^2^, 5000–10,000 m^2^, 10,000–100,000 m^2^, and > 100,000 m^2^, which are labelled small, medium-small, medium, large and extra-large settlements, respectively. On this basis, large and extra-large settlements are selected as the central settlements, and they are used as the scalar to construct Thiessen polygons. The distribution density and range of Thiessen polygons reflect the core area of culture and the radiation area of the central settlements with the greatest cultural impact^25,75,76^.

**Figure 7:**
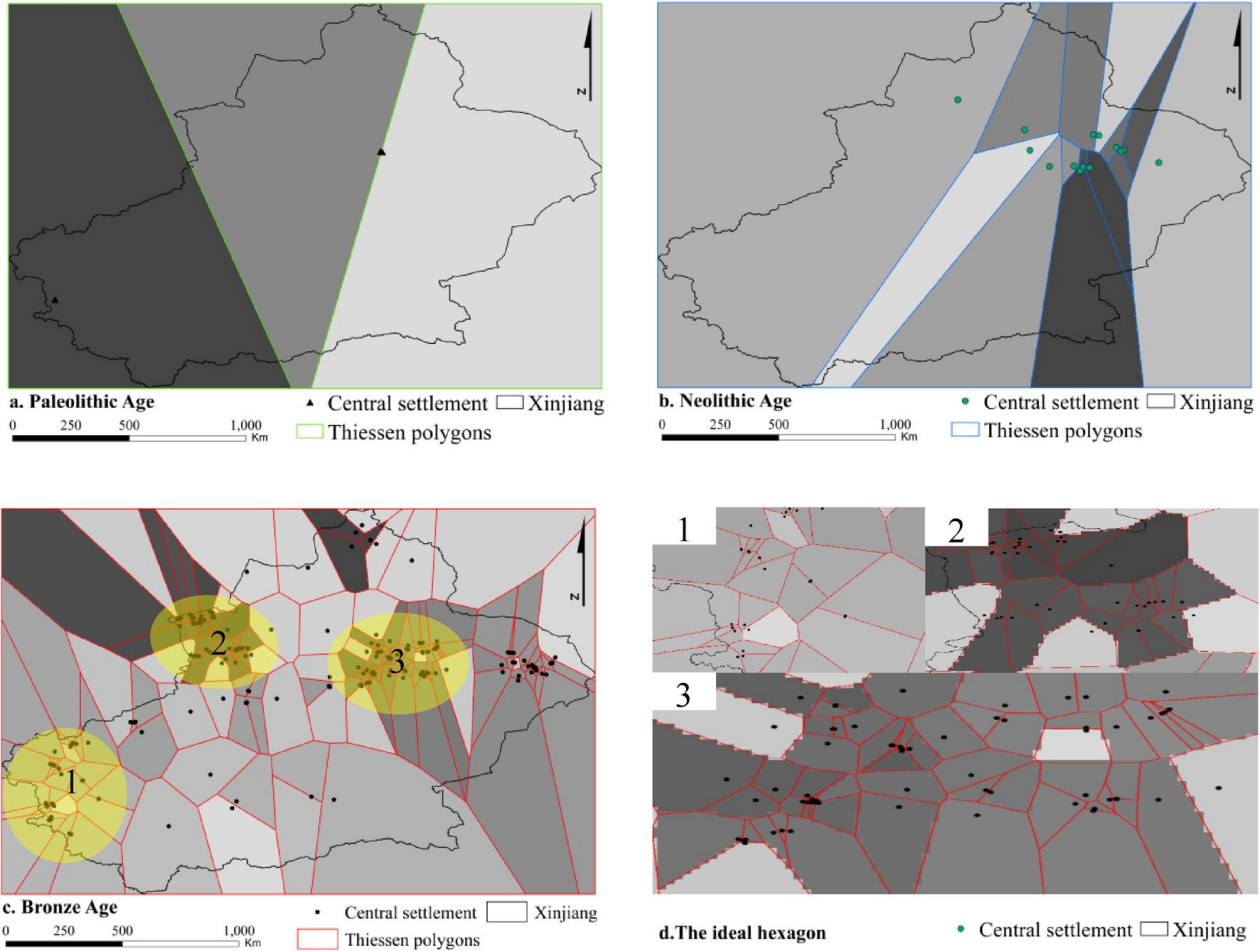
Analysis results of central settlements and Thiessen polygons in each period. Maps are created in ArcGIS 10.2.

According to the classification of settlement size, three central settlements in the Palaeolithic, 14 central settlements in the Neolithic and 320 central settlements in the Bronze Age are obtained. The number of central settlements increases, and the size of the settlements increases. The Thiessen polygon analysis results of each period (Fig. 7a–c) show that with the increase in the number of central settlements, the density of the polygons increases, the scope of settlement activities increases, and several polygonal high-density areas (ellipse delineated areas in Fig. 7a–c) form, which are particularly obvious in the Neolithic and Bronze Ages. The high-density areas are the core areas of cultural development in these later periods. The polygon area CV value of the Palaeolithic is 0.14, that of the Neolithic is 1.68, and that of the Bronze Age is 2.22. According to the definition of the CV value (Duyckaerts and Godefroy^77^), the settlement distribution in the Palaeolithic was in a uniform distribution state. In contrast, because the CV values of the Neolithic and Bronze Age settlement polygons are much greater than 0.64, the distribution is considered significantly concentrated, and the morphology of some polygons is similar to the ideal hexagon mentioned in the centre theory (Fig. 7d). The development of large and extra-large central settlements affected the layout of settlements in surrounding areas.

#### Cultural preference characteristics of settlement distribution

On the basis of quantifying the cultural radiation area of central settlements, the gravity model is used to measure the contact strength of noncentral settlements to central settlements in each polygon. The greater that the gravity value is, the clearer that the preference for culture is, allowing for exploration of the cultural preference characteristics of settlement distribution. The gravity value between noncentral settlements and central settlements in the Palaeolithic period was between 33.0602 and 0.0268, that in the Neolithic period was between 10,540.2431 and 0.0268, and that in the Bronze Age was between 460,689.7756 and 0.0004. The maximum gravity varied significantly, related to the settlement scale being used as the gravity model to measure the data in this paper. The larger that the scale is, the smaller that distance is between central settlements and noncentral settlements, and the greater the gravity that value is. Additionally, it also shows that there are large-scale secondary settlements around the central settlement, and these secondary settlements exist as the appendages of the central settlement and take on some transfer elements of the central settlement. On the one hand, the enhancement of cultural preference reflects the expansion of settlements. On the other hand, the narrowing of the distance between settlements. The narrowing of distance causes settlements to change from a scattered distribution to a concentrated distribution and gradually form settlement groups. The essence of this settlement cluster behaviour lies in the development of productivity. Humans have progressed from fishing and hunting to farming. The food obtained per unit area has increased, and the unit area needed to maintain human survival has decreased; hence, a smaller space can maintain a greater population than before.

The CVs of the gravity values of the settlements in the Palaeolithic, Neolithic and Bronze Age are 2.73, 2.38 and 15.81, respectively. The CV values indicate that the gravity values are concentrated and that the influence of the centrality of the central settlements in the Bronze Age was significant. Hence, the settlement activities had different preferences for culture. Therefore, we used natural fracture classification and found that eight of the nine Palaeolithic gravity values are less than 0.3769, and only one gravity value is 33.0602 (maximum). The reason for this maximum is that the size of the central settlement is the largest in the Palaeolithic (120,000 m^2^), and the distance coefficient from the central settlement is the smallest (0.0778). The spatial connection between the two is the strongest, and settlement activity is the most likely to produce cultural preferences. Whether there is a connection between two settlements is mainly based on whether the unearthed artefacts are similar and whether there is a sequential relationship in time. The range of gravity values in the Neolithic is 10,540.22, which is 9.69 times the average value at that time, and the difference in gravity values is significant. Of the 1277 gravity values for the Bronze Age, 91.46% are less than 100, 6.50% are between 100 and 1000, and the remaining few are between 1000 and 4,606,890.

### Prediction of settlement distribution based on human settlement preference in different periods

**Figure 8:**
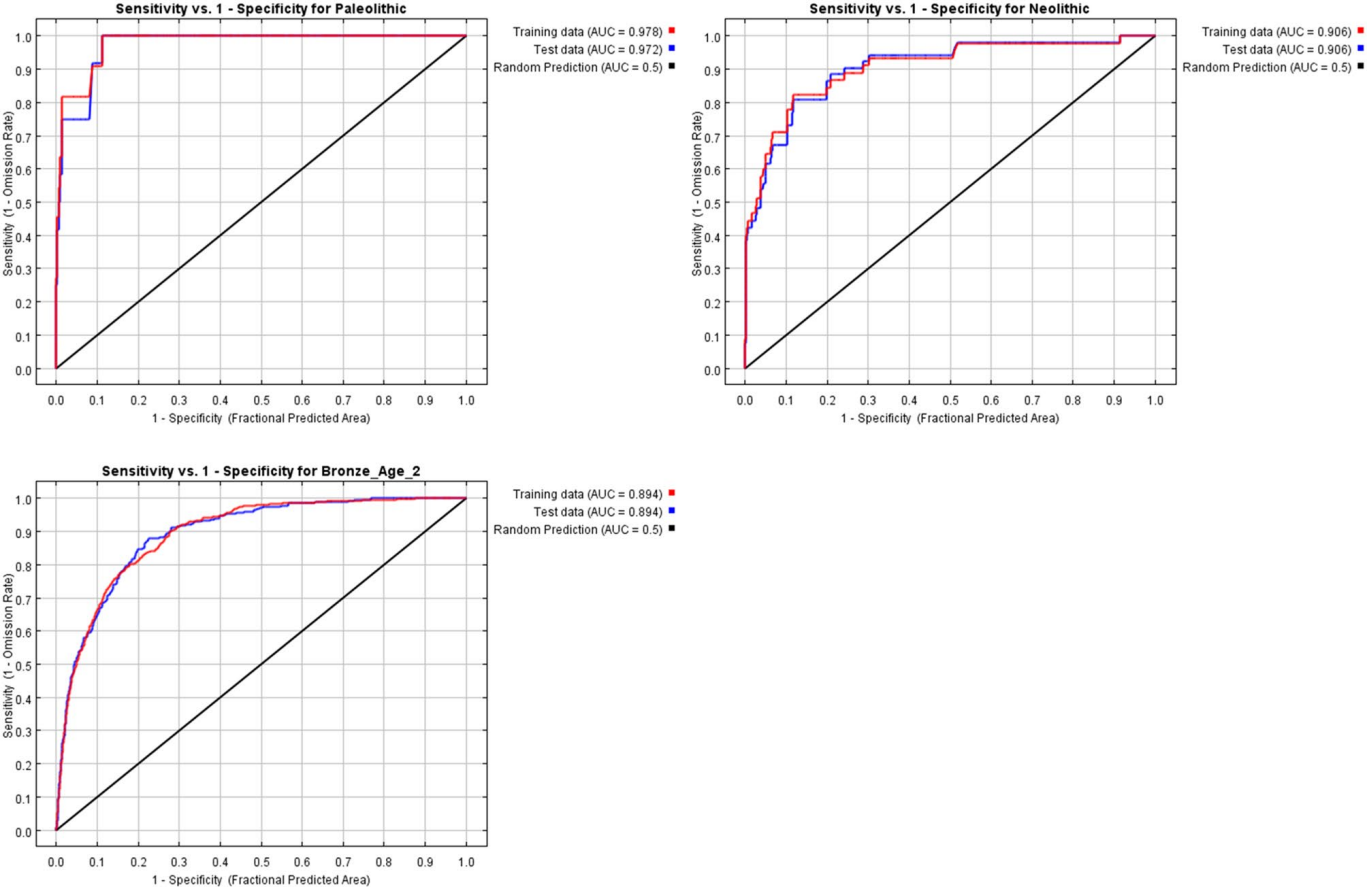
ROC curves of prediction results in different periods.

Based on the above results and previous studies, the settlement environmental variables selected in this paper include slope (x_1_), aspect (x_2_), elevation (x_3_), distance from a river (x_4_), geomorphic type (x_5_), soil type (x_6_), vegetation type (x_7_), profile curvature (x_8_), distance from a traffic route (x_9_), and distance from a central settlement (x_10_). To prevent a strong correlation between environmental variables (which could affect the prediction accuracy), this paper performed correlation analysis (band collection statistics) in ArcGIS software and found that there was a strong correlation between x_9_ and x_4_, with a value of 0.82. Because the early traffic routes are correlated with rivers and other factors and have a certain similarity with the river distribution, x_9_ can basically reflect the preference of settlement activities for rivers, so x_4_ is eliminated and x_9_ is retained. The Maxent model was used to predict the locations of settlements in the Palaeolithic, Neolithic and Bronze Ages. During the prediction process, 30%, 25%, 20%, 15% and 10% of settlements were selected as test data, and 10 iterations were performed. The results with the largest AUC value were selected for comparison of the test data. The results show that, when the proportion of test data is 10%, the AUC value of Palaeolithic settlement prediction is the largest (0.975). When the proportion of test data is 15%, the AUC value of Neolithic settlement prediction is the largest (0.906). When the proportion of test data is 25%, the AUC value of the Bronze Age settlement prediction is the largest (0.895). The ROC curves under the corresponding proportions are shown in Fig. 8. The AUC value of each period model is close to 1, and the model has high accuracy. Therefore, the prediction results less than the above test data proportions are selected as the final prediction results in this paper.

**Figure 9:**
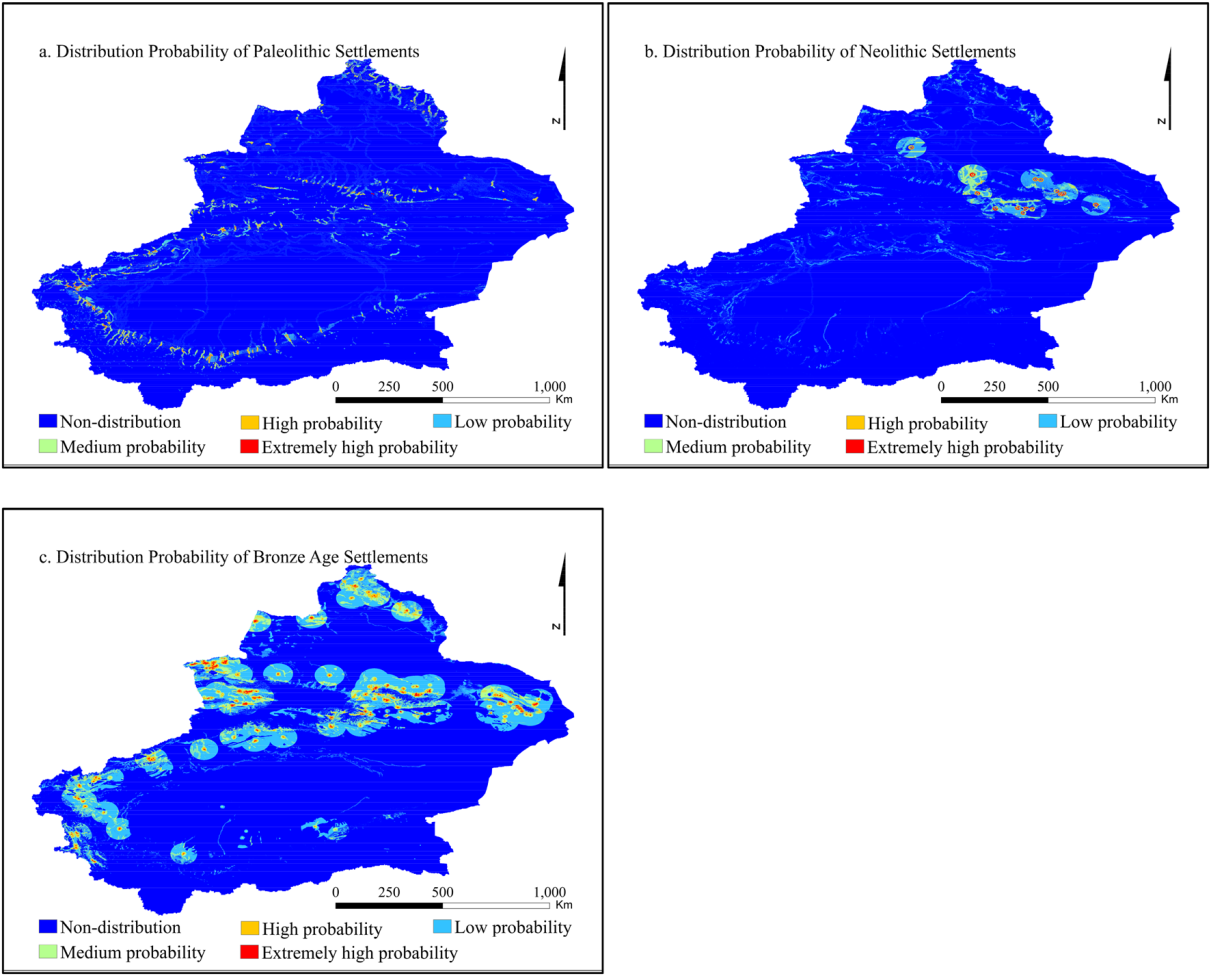
Distribution probability of settlements in different periods. Maps are created in ArcGIS 10.2.

To improve the readability of the results, we reclassify the final prediction results and divide them into five categories using an equal interval distribution: no distribution area, low-probability area, medium-probability area, high-probability area and extremely high-probability area. The results are shown in Fig. 9. The gain values of settlements in the extremely high-probability region in each era are 0.9954, 0.9928 and 0.9875, which are close to 1, and the prediction results are reliable. Based on the statistics of the extremely high-probability area, the area ratios of the extremely high-probability area of settlement distribution from the Palaeolithic to the Bronze Age are 0.12%, 0.10% and 0.29%, and the area of the extremely high-probability area generally gradually expands. From the perspective of distribution area, the extremely high-probability area is mainly distributed in piedmont and intermountain valleys along traffic routes (rivers), including the northern slope of the Tianshan Mountains, the southern slope of the Altai Mountains, the edge of the Tarim Basin, and the Pamir Plateau, where Tashkurgan County is located.

**Figure 10:**
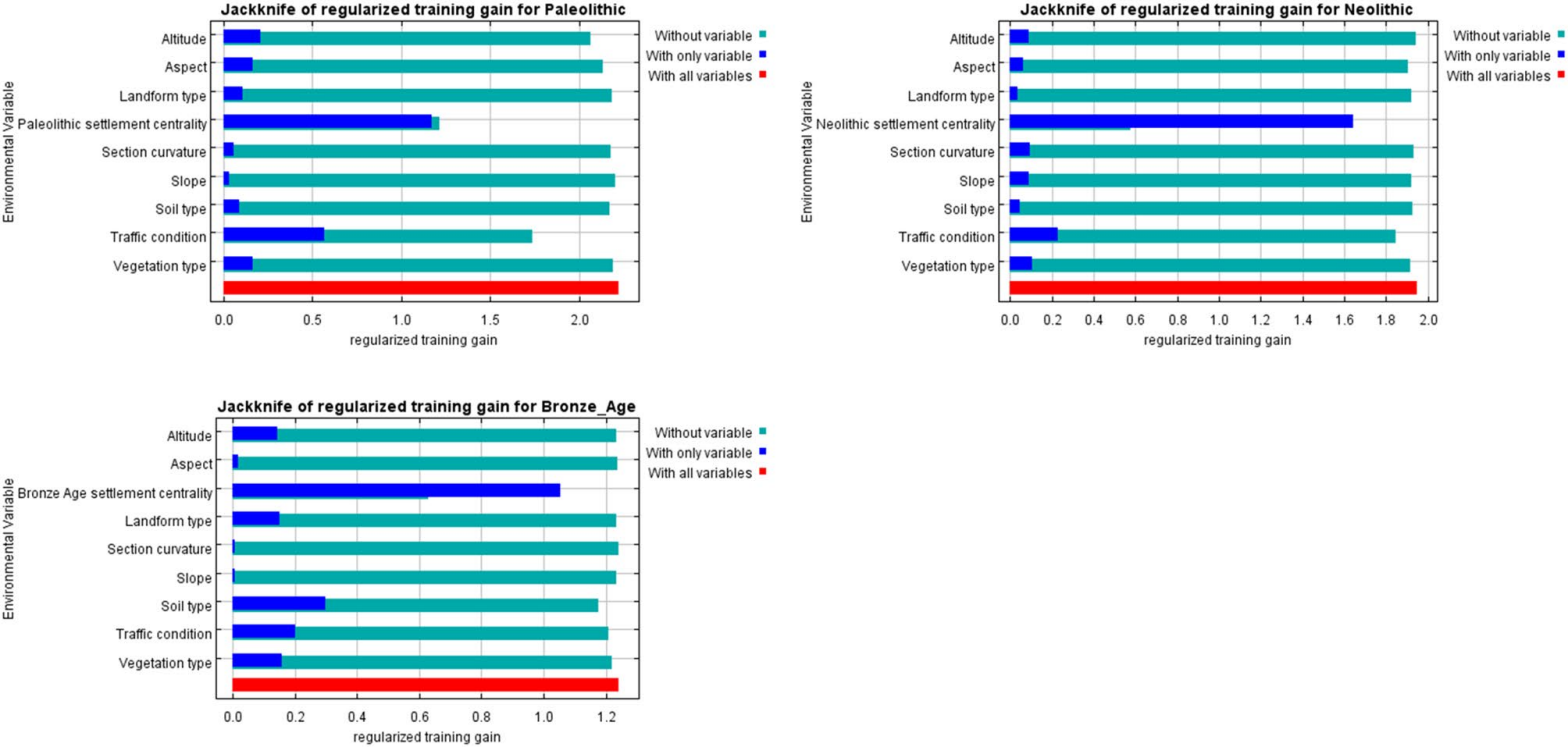
Influence of environmental variables on settlement distribution in different periods based on knife plots.

The contribution rate of environmental variables to the distribution of settlements actually reflects the preference intensity of settlement activities for different environmental variables. The knife plot of the weight of environmental variables (Fig. 10) shows that the preference intensity of settlement activities for different environmental variables varies considerably. For Palaeolithic settlements, the order is centrality (48.48%) > traffic condition (24.16%) > elevation (10.6%) > aspect (4.42%) > vegetation type (3.74%), and the minimum intensity of settlement preference is for slope (0.99%). For Neolithic settlements, the order is centrality (81. 04%) > traffic condition (8.31%) > vegetation type (2.96%) > slope (2.75%) > aspect (2.04%), elevation (0. 07%). For Bronze Age settlements, the order is centrality (75.76%) > soil type (10.45%) > vegetation type (5.43%) > traffic condition (5.17%) > slope (1.34%). Settlements in each period showed a centripetal effect around central settlements, reflecting the production collaboration and cultural identity of early human activities. The influence of central settlements peaked in the Neolithic and then decreased in the Bronze Age. We believe that the limited population and limited productivity in the Palaeolithic were insufficient to form a prosperous production and cultural development state in central settlements. The spatial migration of the population was low, and settlement activities were mostly performed to meet basic survival requirements. The productivity progress and population growth in the Neolithic resulted in rich cultural types. Social organizations developed, and large settlements formed and attracted surrounding settlers. Settlement activities around the central settlements occurred, and settlement clustering increased. The use of metal tools in the Bronze Age resulted in a great increase in the progress of productivity, causing changes in the form and relationship of production. The development of human civilization entered a new stage. The flourishing of culture and production changed the agglomeration of spatial settlement. Social progress, such as cultural pluralism, enhanced quality of life, territorial expansion, growth of the number of large and medium-sized settlements, diversification of demand and establishment of a tribal state system, resulted in a shift in human settlement cultural preferences towards pluralism and reduced the clustering around central settlements.

## Discussion

In this process, there is a close, logical relationship between the influence of natural environmental preferences and social and cultural preference on settlement preference. Appropriate settlements must often meet a variety of preferences. Preference factors affect the choice of settlements through multiple superpositions and interactions, and the interaction of different factors presents different types of enhancement, weakening or mutual independence. Multifactor interaction introduces some difficulties and confusion to the study of settlement preference. However, as a derivative of the human–land system, settlement preferences are not independent of each other. Under the guidance of intangible social culture, human beings transform the physical environment, adapt and construct a suitable natural environment for settlement, and integrate social cultural preferences and natural environment preferences into settlement selection through cross-penetration.

For the early human settlement preference system in Xinjiang, on the one hand, the distribution of mountains and basins in Xinjiang alternates, the climate system is complex, the natural environment has strong differences and inhomogeneity, climate change has occurred in the Holocene^26,27,78^ and the natural environment has changed over time^34,79,80^, resulting in changes in settlement patterns^81,82^. On the other hand, Xinjiang is located in the hinterland of the Eurasian continent and is an important component connecting the Eurasian continent. Spatial corridors, such as the Wahan Corridor, the Ili River Valley, the Alashankou and the Hexi Corridor, provided good conditions for early human migration and cultural exchange in Xinjiang^31^; thus, the early human culture in Xinjiang was diverse, as evidenced by the presence of settlement technologies originating in Moscow and Russian Siberia^41,83^ and the Linya and Yanbulake cultures with cultural characteristics similar to those found on the Central Plains of China^47,48,84,85^. Hence, the diversified cultural types and environmental evolution rendered the early human settlement preferences in Xinjiang quite different.

A previous study found that Neolithic settlements exhibited a clear preference for low elevations, but the upper limit of the elevation distribution of the settlements was greater than that of the Palaeolithic and Bronze Age. From the Palaeolithic to the Neolithic, there was also a large increase in the number of settlements and a decrease in the influence of elevation on the distribution of settlements because of the low productivity in the Palaeolithic and the warm and humid climate in the Neolithic in Xinjiang. The climate was more suitable for survival in the late Holocene^35,36,86,87^ than in the last glacial period. Especially when compared to the Palaeolithic period conditions under the influence of Younger Dryas impact hypothesis^28,88^, the Neolithic moisture and temperature conditions were superior and were conducive to human space expansion and settlement. The cold and dry climate (4.3–3.8 ka BP, 4.3 ka event) and its lagged influence on the early late Holocene in Xinjiang restricted the development of the Bronze Age in Xinjiang to some extent^89–91^. This climatic influence is also an important reason why the maximum elevation of settlements in the Bronze Age was 814 m lower than that during the Neolithic and why the settlement preference for rivers was different from that in the Neolithic, as evidenced by the decline of the Xiaohe Culture in Xinjiang^91^.

Since the Holocene, there has been extensive dissemination and exchange of culture, technology and means of production in Xinjiang^31^, thereby providing conditions for the progress of civilization in Xinjiang and promoting the transformation of the early human livelihood model in Xinjiang against the background of environmental evolution, from livestock fishing–hunting economy, to a mixed mode of farming and animal husbandry^32,92,93^. Combined with the settlement preference intensity of the natural environment (Fig. 10), the strong elevation and slope preferences in the Palaeolithic period were the responses of settlement activities to the human settlement temperature demand in the cold and dry climate at the end of the Palaeolithic period. The strong vegetation type preferences in the Palaeolithic and Neolithic eras were the embodiment of the production needs of the hunting and gathering livelihood mode. In the Bronze Age, productivity increased, and the climate tended to be stable. Livelihood patterns dominated by farming agriculture and animal husbandry formed in Xinjiang^32,92^, and the demand for agricultural soil and vegetation for animal husbandry forced the settlement distribution to expand into areas with appropriate soil and vegetation types, which explaining the strong preference of human settlement for soil and vegetation types in the Bronze Age. Therefore, the early human livelihood model was not only a component of the settlement preference system but also a driving factor, and it had a clear impact on settlement preference.

Although the Palaeolithic to Bronze Age in Xinjiang experienced cold to warm and dry to wet climatic changes, causing changes in the natural environment, the expansion of the spatial distribution of settlements and the diversification of settlement preferences show that with the progress of productivity, human cognition and growth-enhancing behaviours, the ability of settlements to adapt to the changes in and transform the natural environment improved, and the dependence of settlement activities on natural environmental conditions decreased. The cultural preference characteristics of settlement distribution and the influence of the centrality of central settlements on settlement distribution (Fig. 10) show that from the Palaeolithic to the Bronze Age, the increase in settlements, the scope of human activities and the enhancement of human adaptation and transformation ability gradually influenced the spatial distribution of human settlements, strengthened the spatial connection between settlements, and generated settlement cluster behaviours, providing conditions for the birth and development of a group cultural system. The centrality preference of settlements around central settlements increased, the cultural preference of settlement activities was enhanced, and human initiative was fully exerted. The late Holocene was the peak of human activities in Xinjiang. Population growth and settlement expansion were intense, and human migration was frequent. The influence of traffic conditions on settlement distribution increased, and the intensity of traffic settlement preference was at a high level. Thus, human adaptation and transformation are the main factors controlling the settlement distribution characteristics, reflecting the great role of human initiative. This strong initiative reflects not only the enhancement of ancient human self-awareness and the strengthening of communication between people but also the manifestation of the progress of human civilization^94^.

## Conclusion

Early human settlement activities in Xinjiang showed different preferences for natural environmental and social environmental conditions in different periods. The dependence of settlement activities on natural environment conditions decreased, and the natural environment preferences tended to be diversified, while the preferences for traffic conditions tended to decline. However the cultural preferences of early human settlements increased as human settlements expanded, and settlements showed a preference for spatial agglomeration.

As productivity and social progress increased, social settlement preferences tended to diversify, reflecting the increased adaptability of early humans to the environment. Climate change and livelihood patterns had significant impact on settlement preferences as settlement activities adapted to and remodelled changes in the natural environment. The cultural preference for settlement activities was strong, and the human initiative factor was the main controlling factor. Driven by this cultural preference, settlement activities shifted from a scattered distribution to a systematic cluster distribution, and the settlement system gradually formed. We believe that settlement needs and environmental conditions gradually formed a relatively stable and progressive interactive relationship in the process of adaptation and transformation.

Finally, based on the prediction of the distribution of early human settlements based on the characteristics of settlement preference, it was found that the distributions of the high-probability areas of settlements and existing settlements were basically concentrated in areas with mild conditions, notably in the foothills and valleys. For settlement prediction in different periods, Maxent performs well in terms of practicability, but the proportion of test data affects the prediction accuracy. Therefore, it is necessary to use trial and error in the prediction process to select the appropriate proportion of test data.

## Materials and methods

### Data

The site data used in this study are mainly from many years of archaeological excavations in Xinjiang. Based on publications such as “China Cultural Relics Atlas · Xinjiang Volume”, “A compilation of cultural relics and archaeological materials in Xinjiang”, “China Statistical Yearbook on Archaeology” and the third national cultural relics survey results, site data in the study area were collected, and the sites with unknown dates were eliminated. A total of 1658 settlements are retained (Fig. 1), including 12 sites of the Palaeolithic, 52 sites of the Neolithic and 1594 sites of the Bronze Age (Table S1). In this study, the name, longitude and latitude coordinates, cultural relic category, age, time, area, protection level and other information on the site were collected, and GIS data on the sites were obtained through ArcGIS vector transformation. Due to the lack of enough reference for the spatial data set of existing sites in this area, there may be some spatial position errors in the data. The spatial position error should be between 0 and 1 km. However, from the perspective of data application, this data is more used for macro analysis on a large spatial scale than the discussion of microsite information requiring high-precision location information. From this point of view, the spatial error of sites is understandable.

Digital elevation model (DEM) elevation data with a resolution of 30 m were obtained from the geospatial data cloud website (http://www.gcloud.cn), and 1:1 million-scale vegetation type spatial distribution data for China were obtained from the Chinese Academy of Sciences Resource and Environmental Science Data Center (http://www.resdc.cn/Default.aspx)^95^. Chinese soil attribute data and landform type data come from the National Qinghai-Tibet Plateau Science Data Center (http://data.tpdc.ac.cn)^56,96^. Based on the above data, ArcGIS10.2 software was used to extract and produce data on the elevation, slope, water system, slope, slope direction, soil type and landform type for each site. GIS and archaeological spatial analysis methods were further used to explore the environmental preference characteristics of settlement distribution from the Palaeolithic to the Bronze Age in Xinjiang.

### Traffic route simulation based on GIS

The more primitive that the era of information development is, the more significant that the impact of traffic conditions is on cultural transmission. The routes of migration and communication are also proof of human activities, and human settlement activities are bound to be distributed along traffic routes. Therefore, the screening of early traffic routes has a good indicative effect on the study of the distribution of human settlement sites, which is of great significance for the study of early human settlement prediction. Due to the lack of written records, high-precision map records and road sites, the early traffic routes in most regions remain unclear. Although we can deduce the early lines from the existing settlement sites, this inversion is bound to be affected by the excavation of settlement sites. The traffic routes in the settlement distribution areas that have completely disappeared and have not been excavated cannot be effectively identified, and the accuracy of traffic route identification is affected. Therefore, it is necessary to use the natural environmental conditions that affect the distribution of settlement sites as simulation factors to identify traffic routes as comprehensively as possible. Early human settlement was affected by strong geographical conditions, such as rivers, slopes, the elevation and other factors^16,25,50,59,60^. Referring to Zhu et al.’s simulation method of early human traffic routes on the Qinghai-Tibet Plateau^50^, this paper uses ArcGIS hydrological analysis and other tools to simulate the traffic routes in Xinjiang to analyse the impact of traffic factors on settlement activities and to interpret the traffic preferences of early human settlement.

### Settlement cultural radiation range

Thiessen Polygons is a space segmentation method that seamlessly divides the plane space according to the known point set. The method is widely used in various spatial influence analyses related to distance^25,75,76^. In this study, by extracting large and medium-scale settlements as the central settlement, taking the central settlement as the generator, and the settlement area as the weight, Thiessen polygons were used to determine the spatial influence range of the central settlement generator, which was used as the cultural radiation area of the central settlement.

### Establishment and test of the settlement prediction model

The Maxent model is the realization of the maximum entropy theory. When the known conditions are met, no biased assumptions are made about the unknown situation to obtain the prediction results with the minimum risk^97^. In this case, the geographical distribution of this landscape can be predicted and described according to the correlation between a certain landscape location and environmental variables, with good applicability in site prediction^98–100^. In this paper, the maximum entropy distribution is obtained using the settlement data as a constraint condition and the environmental impact background of the settlement distribution as a variable^101,102^. The prediction results of the model were tested by the area under the curve (AUC) of the receiver operating characteristic curve (ROC). The range of the AUC was [0, 1]. The accuracy of the model was low when the AUC was 0.5–0.7, high when the AUC was 0.7–0.9, and the highest when the AUC was greater than 0.9^61,103^. Kvamme’s gain statistics were used to evaluate the accuracy of the prediction results^104^, and the prediction results with gain values closer to 1 were used as the final results. The final results were loaded into ArcGIS software to visualize the settlement distribution probability map (Fig. 9).

## Supplementary Information

Supplementary Legends.

Supplementary Information.

Supplementary Table S1.

## Data Availability

The data of this research has been released in the National Qinghai-Tibet Plateau Data Center (http://data.tpdc.ac.cn), https://doi.org/10.11888/HumanNat.tpdc.271910. The website is http://data.tpdc.ac.cn/en/disallow/bb49a6da-bfd4-4355-9d0c-988eef793ee1/.
