# Supplementary material for: The study of early human settlement preference and settlement prediction in Xinjiang, China
Source: Sci Rep. 2022 Mar 24;12:5072. doi: 10.1038/s41598-022-09033-y (PMC8948180; doi:10.1038/s41598-022-09033-y)
Supplement: Supplementary file 1 — Supplementary Legends. [file 41598_2022_9033_MOESM1_ESM.docx]

**Captions for Supplementary Materials**

Supplementary Material 1: Data information description

Supplementary Table 1: Site distribution and geographical environment background data. Contains the site location information used in this study and the extracted modern geographic environment information.
