# Supplementary material for: The study of early human settlement preference and settlement prediction in Xinjiang, China
Source: Sci Rep. 2022 Mar 24;12:5072. doi: 10.1038/s41598-022-09033-y (PMC8948180; doi:10.1038/s41598-022-09033-y)
Supplement: Supplementary file 2 — Supplementary Information. [file 41598_2022_9033_MOESM2_ESM.docx]

**Supplementary Material 1:**

**Data information description**

**Spatial distribution data of cultural sites from the Paleolithic to Bronze Age in Xinjiang**

Bo Tan^1,2^, Hongwei Wang^1,2^, Xiaoqin Wang^1,2^, Suyan Yi^1,2^, Jing Zhou^1,2^, Chen Ma^1,2^, Xinyan Dai^1,2^

^1^Key Laboratory of Oasis Ecology, Xinjiang University, Urumqi 830046, China

^2^Key Laboratory of Smart City and Environment Modelling of Higher Education Institute, College of Resources and Environment Sciences, Xinjiang University, Urumqi 830046, China

The site data used in this study are mainly from many years of archaeological excavations in Xinjiang. Based on publications such as “China Cultural Relics Atlas · Xinjiang Volume”, “A compilation of cultural relics and archaeological materials in Xinjiang”, “China Statistical Yearbook on Archaeology” and the third national cultural relics survey results, site data in the study area were collected, and the sites with unknown dates were eliminated. In this study, the name, latitude and longitude coordinates, site type, civilization and cultural age, time elapsed, area, altitude, slope, aspect, soil type, vegetation type, landform type and other information on the site were collected.

Digital elevation model (DEM) elevation data with a resolution of 30 m were obtained from the geospatial data cloud website (http://www.gcloud.cn), and 1:1 million-scale vegetation type spatial distribution data for China come from the Chinese Academy of Sciences Resource and Environmental Science Data Center (http://www.resdc.cn/Default.aspx). Chinese soil attribute data come from the National Qinghai-Tibet Plateau Science Data Center (http://data.tpdc.ac.cn/zh-hans/), and Chinese landform type data come from the geographical conditions detection cloud platform (http://www.dsac.cn/). Based on the above data, ArcGIS10.2 was used to extract and produce data on elevation, slope, water system, slope, slope direction, soil type and landform type for each site.

The data set of this research has been released in the National Qinghai-Tibet Plateau Data Center (<http://data.tpdc.ac.cn>). DOI: 10.11888/HumanNat.tpdc.271910. The website is <http://data.tpdc.ac.cn/en/disallow/bb49a6da-bfd4-4355-9d0c-988eef793ee1/>. Cite as: Tan, B. (2021). Spatial distribution data of cultural sites from the Paleolithic to Bronze Age in Xinjiang, China. National Tibetan Plateau Data Center, DOI: 10.11888/HumanNat.tpdc.271910. CSTR: 18406.11.HumanNat.tpdc.271910.
